# Carbonic anhydrase IX expression is more predictive than prognostic in breast cancer

**DOI:** 10.1038/sj.bjc.6603715

**Published:** 2007-03-27

**Authors:** P N Span, J Bussink, P H De Mulder, F C G J Sweep

**Affiliations:** 1 Department of Chemical Endocrinology, Radboud University Nijmegen Medical Centre, PO Box 9101, Nijmegen 6500 HB, The Netherlands; 2 Department of Radiation Oncology, Radboud University Nijmegen Medical Centre, PO Box 9101, Nijmegen 6500 HB, The Netherlands; 3 Department of Medical Oncology, Radboud University Nijmegen Medical Centre, PO Box 9101, Nijmegen 6500 HB, The Netherlands

**Sir**,

In a recent paper in the *British Journal of Cancer*, Hussain *et al* (2007) found carbonic anhydrase IX expression to be related to a poor prognosis in 144 unselected breast cancer patients. They state: ‘CA IX expression may contribute to the identification of patients at greater risk of relapse who should be offered adjuvant treatment while sparing those whose prognosis is already good.’

However, in their patient description, Hussain *et al*. do not give any information concerning whether adjuvant treatment was given to these patients and, if so, which type of treatment. This makes the data difficult to interpret. We have earlier described the interactions of CA IX expression with both adjuvant endocrine and chemotherapy in 253 breast cancer patients (Span *et al* 2003). CA IX is induced by hypoxia inducible factor (HIF)-1, and is particularly overexpressed in VHL-related clear-cell renal carcinoma. Hypoxia itself is known to be associated with a poor response to radio- and chemotherapy. This indicates that CAIX expression is not prognostic – being associated with disease progression irrespective of treatment – but rather predictive, –predicting which patients will benefit from adjuvant treatment and who will not.

As such, we disagree with the authors' statement that CA IX expression will identify patient who should be offered adjuvant treatment due to a poor prognosis. Rather, more aggressive treatment or alternative treatment modalities might be offered to patients with high CA IX expression in their breast tumours, as the available data indicate that radiotherapy, endocrine and chemotherapy will be less successful for them than for patients with low CA IX expression.
